# A gapless genome assembly of a *Japonica* variety ‘BD8’ provides insights into rice salt tolerance

**DOI:** 10.3389/fpls.2025.1713117

**Published:** 2025-12-12

**Authors:** Leiyue Geng, Tuo Zou, Wei Zhang, Shuo Wang, Haining Wang, Guangsheng He, Qi Du

**Affiliations:** 1 Institute of Coastal Agriculture, Hebei Academy of Agriculture and Forestry Sciences, Tangshan, Hebei, China; 2 Tangshan Key Laboratory of Rice Breeding, Tangshan, Hebei, China

**Keywords:** rice, genome assembly, telomere-to-telomere, salt-tolerant, transcriptome analysis

## Abstract

**Background:**

Accurate genomic information in specific rice varieties is essential for functional gene mining and advancing molecular breeding. Bindao8 (BD8) is an elite Japonica rice variety with notable salt tolerance.

**Results:**

This study presents a near telomere-to-telomere (T2T) genome assembly of ‘BD8’, with a size of 384.2 Mb and contig N50 of 31.69 Mb. A total of 58,685 genes were identified, 95.93% (56,297) of which were functionally annotated. Phylogenetic analysis clarified the genetic background of this cultivar. Abundant structural variations were detected, and enrichment analysis of unique genes identified four enriched KEGG pathways, including amino sugar and nucleotide sugar metabolism (ko00520), which supplies UDP-glucose/UDP-glucuronate for cell-wall biosynthesis. To address BD8’s salt tolerance, time-course transcriptomic profiling was integrated with the genome, revealing two salt-specific gene clusters (347 and 607 genes) and five significantly enriched salt-responsive KEGG pathways. Notably, the amino sugar and nucleotide sugar metabolism pathway (ko00520) was commonly enriched in both BD8’s unique genes and salt-specific clusters, suggesting that cell-wall precursor supply is a constitutive and inducible component of BD8’s tolerance mechanism.

**Conclusion:**

The high-quality BD8 genome assembly will serve as a critical resource for rice functional genomics and genomics-driven molecular breeding. The integration of BD8’s genome with transcriptomic data highlights the amino sugar and nucleotide sugar metabolism pathway as a key target for improving salt tolerance in rice breeding programs.

## Introduction

Rice (*Oryza sativa* L., 2n=2x=24) is a staple crop feeding approximately half of the world’s population (Elert, 2014). Rice genome is relatively smaller than other monocotyledonous species, hence it usually serves as model species for monocot and crop plant research (Shang et al., 2023). High-quality reference genome assemblies are essential for crop improvement and provide unprecedented opportunities to understand important agronomy traits, deploy technologies to introduce genetic variation, and assist for precision molecular breeding (Varshney et al., 2020). During the last two decades, reference genomes of a multitude of plant species have been reported using different sequencing technologies, and rice (*O. sativa* L.) genome was the first crop genome to be assembled (Garg et al., 2024). In 2005, the International Rice Genome Sequencing Project (IRGSP) published the first Sanger-sequencing-based rice reference genome of Nipponbare (*Oryza sativa subsp. japonica*, Nip) (Goff et al., 2002), and it was a major milestone in the field of plant genomics (International-Rice-Genome-Sequencing-Project, 2005). Since then, advances in genome sequencing and assembly methodology, multiple high-quality genomes of Indica and Japonica subspecies have been assembled, annotated and released, including IR64, R498, Zhenshan 97, Minghui 63, Taichung Native1, LTH, Kitaake, IR8, N22, Huajingxian74, HR12, Basmati 334, Dom Sufid, Huazhan and Tianfeng etc., at the chromosome level, and Shennong265, DJ123, WR04-6, Suijing18, Koshihikari, Basmati, Kongyu-131, and Guangluai-4 etc., at scaffold level (https://www.ncbi.nlm.nih.gov/datasets/genome/). The quality of these reference genomes varies significantly from draft genomes with hundreds of unplaced scaffolds to nearly finished haplotype phased chromosome-length assemblies.

A high-accuracy telomere-to-telomere gap-free assembly is always the ultimate aim of genome assembling (McCartney et al., 2022). Those assemblies could provide a complete and precise coordinates of plant genome (Benevenuto et al., 2019), including the most complex, highly repetitive regions (telomeres, centromeres, and nuclear-organizing regions) and have important implications in downstream genome-based studies such as evolution and identification of genes/variations associated with specific traits (Li and Durbin, 2024). However, many genome assemblies often contain gaps, on account of limitations of sequencing technology and intricate genomic organization (Jiao and Schneeberger, 2017). The recent advances in long-read sequencing technology, such as Bio-nano (Michael and Buren, 2020), Oxford Nanopore Technologies (Logsdon et al., 2022), and PacBio High-fidelity sequencing (HiFi) (Mostovoy et al., 2016), which produces higher contiguity reads without overlapping and error correction, had significantly improved the quality and continuity of genome assembly. The utilization of long-read technology, and advancement in assembly algorithms, enables the generation of telomere-to-telomere gap-free chromosome level assemblies (Deng et al., 2022; Huang, 2023). It is no longer a daunting task to construct telomere-to-telomere assembly.

With long-read sequencing technology, several rice genomes had been assembled into gapless chromosomes with only 2–5 telomeres absent (Li et al., 2021; Song et al., 2021; Zhang et al., 2022). Especially, Shang et al. (2023) had already produced a complete assembly of the rice reference genome, NIP-T2T (version AGIS-1.0), within which all 12 centromere and 24 telomere regions were resolved. As rice cultivars, landraces and wild rice varieties with dramatically different genetic backgrounds, a single genome inadequately reflects the genomic diversity of an entire species, and there is a growing need for *de novo* genome assembly for special purposes in rice research (Zhao et al., 2018). The genomes of native rice varieties in North China, which is one of the important rice production areas in China, have not been assembled. Bindao8 (*BD8*) is an elite Japonica rice variety (Zhang et al., 2021), which bred by Institute of Coastal Agriculture, Hebei Academy of Agriculture and Forestry Sciences, and had been widely planted in North China Region, and evaluated as the leading variety in Beijing-Tianjin-Hebei region (http://nync.hebei.gov.cn/html/www//tzgg/1737310967762956290.html). It exhibited high yield, widely environmental adaptability and salt tolerant in especial during many years of production and cultivation (https://www.ricedata.cn/variety/varis/623277.htm, **Supplementary Figure S1**). However, the underlying genetic mechanism of BD8 has not been fully revealed owing to the lack of high-quality genome. A high-quality genome of BD8 will fulfill such a need for functional genomics studies and facilitate deciphering its genetic mechanism of salt tolerance. Hence, we launched the high-quality *de novo* assembly project of BD8.

In this study, we successfully produced a gap-free genome assembly of *Japonica* rice variety BD8, with nearly intact centromeres and telomeres. This genome sequence would improve our understanding about the genetic basis of modern high-yielding variety, and assist to identify specific candidate genes or regions of BD8 genome, and will facilitate subsequent genetic breeding practices. We also designed transcriptomic sampling experiments at different time points after salt stress to explore BD8’s transcriptional expression response to salt stress. The genome assembly and annotations will serve as a valuable resource for studying rice genomics and genetics and accelerating rice improvement.

## Materials and methods

### DNA extraction, library construction, and genome sequencing

Genomic DNA was extracted from young leaves of BD8 using a modified CTAB method. DNA quality was assessed via NanoDrop spectrophotometry, Qubit Quantitation, and 1% agarose gel electrophoresis. Short-read libraries (300–700 bp insert size) for Illumina sequencing (paired-end 2×150 bp, HiSeq™ 2500 platform) were constructed using the NEB Next^®^ Ultra™ DNA Library Prep Kit. PacBio HiFi libraries (25 kb subreads) were prepared with the Sequel II platform, and ONT Ultralong libraries (Ligation Sequencing Kit) were sequenced on the PromethION 48 platform.

### RNA-seq sample preparation and sequencing

For RNA-seq, 28-day-old BD8 seedlings were transplanted to salt-tolerant evaluation tanks. After recovery, salt stress was initiated by adding 10 ms/dm saline water. Leaf samples were collected at four salt treatment time points (1 h: initial stage; 12 h: early stage; 48 h: medium stage; 96 h: late stage) and a corresponding control group (seedlings under normal water conditions, sampled simultaneously), with three biological replicates per group. Total RNA was extracted using the RNA Easy Fast Plant Tissue Kit (TIANGEN), and RNA-seq libraries (350 bp insert size) were sequenced on the Illumina HiSeq™ 2500 platform.

### Genome *de novo* assembly and quality assessment

Genome Survey: Illumina raw reads were filtered with fastp (parameters: -q 20 -u 10 -n 5) (Chen et al., 2018). Genome size and heterozygosity were estimated using Jellyfish v2.3.0 (k-mer = 21, -t 1 -s 5 G -C) (Koren et al., 2017) and GenomeScope2 (Vurture et al., 2020).

Assembly: PacBio HiFi reads and Nanopore ONT reads were assembled to generate the contigs with HiFiasm v0.19.5-r587 (parameters: -o BD8.asm –primary -t 48) (Cheng et al., 2021). Hi-C data (processed via Hi-C Pro, https://github.com/nservant/HiC-Pro.git) were used to scaffold contigs into chromosomes with Lachesis (Burton et al., 2013), followed by orientation correction against Nip-T2T using RagTag (Alonge et al., 2022). The final genome assembly is deposited at NGDC (Accession: GWHFWBB00000000.1).

Quality Assessment: Completeness was evaluated with BUSCO (embryophyta_odb10 database) (Manni et al., 2021). Contig N50, Qv (Merqury, Rhie et al., 2020), LAI (LTR assembly index, Ou and Jiang, 2017), and read mapping rates (Minimap2 (Li, 2018) for short reads, VerityMap (Mikheenko et al., 2019) for long reads) were calculated to assess continuity and accuracy.

### Identification of centromere and telomere sequences

Centromere sequences were identified via homologous searches using the 155 bp rice CentO satellite sequence (Cheng et al., 2002). Telomere repeats (5’-AAACCCT-3’ and reverse complement) were detected with TIDK (https://github.com/tolkit/telomeric-identifier) and FindTelomeres (https://github.com/JanaSperschneider/FindTelomeres).

### Genome annotation

Repeat Annotation: RepeatModeler2 (Flynn et al., 2020) and RepeatClassifier (Dfam v3.5) (Bao and Eddy, 2002) were used for ab initio prediction. LTR_FINDER (Xu and Wang, 2007) and LTRharvest (v1.5.9) (Ellinghaus et al., 2008) identified LTR-RTs, filtered by LTR_retriever (v2.8) (Ou and Jiang, 2017). RepeatMasker (v4.1.2) (Tarailo and Chen, 2009) masked transposon sequences with Repbase library (Bao et al., 2015).

Gene Prediction: Integrative annotation combined ab initio (Augustus (Stanke et al., 2008), SNAP (Korf, 2004)), homology-based (GeMoMa Keilwagen et al., 2016, using 9 rice genomes: *O. sativa02428, O. sativa 9311, O. sativaIRGSP-1.0, O. sativa Kosh, O. sativa Lemont, O. sativa MSU7, O. sativa R498, O. sativaT2T, O. sativa ZH11* (**Supplementary Table S10**)), and RNA-seq evidence (Trinity-assembled transcripts, Grabherr et al., 2013). EVM v1.1.1 (Haas et al., 2008) integrated models, and PASA (Haas et al., 2003) refined annotations.

Functional Annotation: BLASTP (E-value ≤ 1e-5) was employed to align proteins against the NCBI Non-Redundant (https://blast.ncbi.nlm.nih.gov), TrEMBL(www.uniprot.org), SWISS-PROT(http://www.uniprot.org). Sequences ID were submitted to EggNOG (http://eggnog-mapper.embl.de), GO (http://geneontology.org) and KEGG databases (https://www.kegg.jp) to assign orthologous group annotations and associated functional terms. Protein Families domains (https://pfam.xfam.org) were identified using InterProScan with HMMER (Jones et al., 2014) for conserved domain analysis. Noncoding RNAs were predicted using tRNAscan-SE (tRNA, Lowe and Eddy, 1997)), Barrnap (rRNA, Loman, 2017), Infernal (snRNA/miRNA, Nawrocki and Eddy, 2013), and GenBlasta (pseudogenes, She et al., 2008).

### Synteny and comparative genomic analysis

Synteny: MUMmer4 (Marçais et al., 2018) aligned BD8 and Nip-T2T genomes (parameters: -mum -l 40 -c 100). Delta-filter (parameters: -i 90 -l 500 -1) and SyRI (Goel et al., 2019) identified SNPs, InDels, and structural variations.

Phylogenetics: OrthoFinder v2.4 (diamond, e-value 0.001; Emms and Kelly, 2019) clustered protein sequences from 15 species (**Supplementary Table S10**). Single-copy orthologs were aligned with MAFFT (parameters: -localpair -maximal 1000, Mikheenko et al., 2019), trimmed with gBlocks (Robert, 2004) (parameters: -B5 = h), and used to construct a maximum likelihood tree with IQ-TREE v2.1.4 (JTT+F+R5 model, 1000 bootstraps, Nguyen et al., 2015).

### Salt-responsive DEG identification

RNA-seq reads were mapped to the BD8 genome using Hisat2 (Kim et al., 2019). Read counts and TPM values (transcript per million, Wagner et al., 2012) were calculated with feature Counts and DESeq2 (Love et al., 2014). DEGs were defined as |log_2_Fold Change| ≥ 2 and FDR < 0.05. To classify these DEGs to salt tolerance in BD8, salt-responsive genes were identified by comparing salt-treated samples with their corresponding controls for each cultivar. Genes showing significant expression changes between the control and salt stress were considered as salt-responsive genes.

Dynamic transcriptomic responses to salt stress were analyzed using STEM (Ernst and Bar-Joseph, 2006). Gene expression trends were clustered into 20 profiles, with significance defined by p-value < 0.05 and fold change > 2. Profiles showing significant divergence between salt-treated and control groups were retained, and their constituent genes were subjected to KEGG pathway enrichment via KOBAS (Xie et al., 2011).

## Results

### Genome sequencing and assembly of the high quality BD8 genome

Long-read sequencing platforms including Oxford Nanopore and Pacific sequel II combining with short-read platforms including Illumina and Hi-C sequence were applied to *de novo* the high-quality genome of BD8. As a result, a total of ~ 20.41 Gb (53.11 X) ONT long reads, ~ 23.35 Gb (60.76 X) HiFi reads, ~20.54 Gb (53.11 X) paired-end (PE) Illumina short reads, and ~ 45.09 Gb (117.33X) Hi-C reads were generated after filtering, respectively (**Supplementary Table S1**).

**Figure 1:**
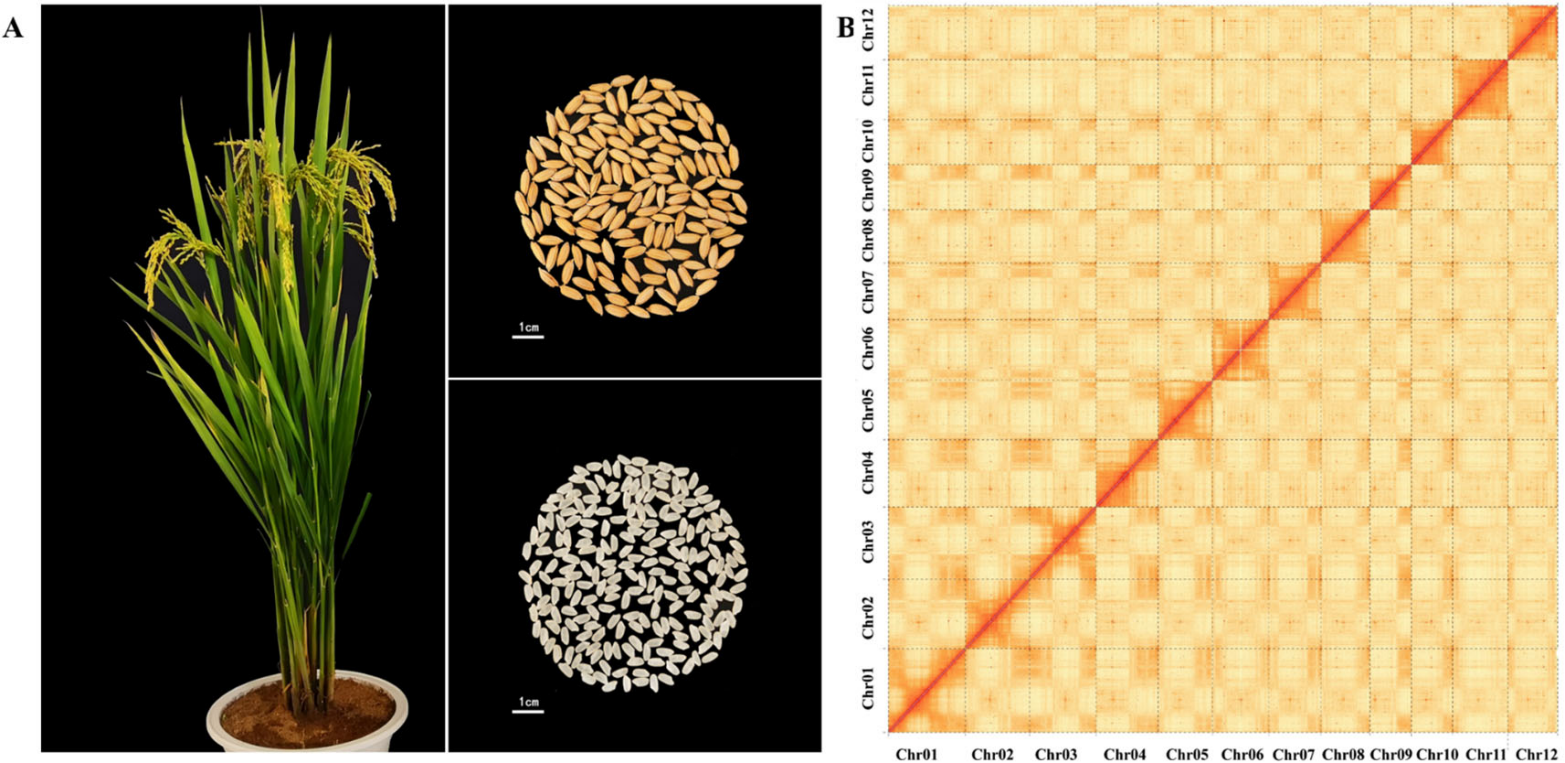
Overview of the *BD8* genome. **(A)** Growth, grain and rice of BD8. Bindao 8 is a long-grain variety, with tough stalks, high lodging resistance, upswing plant shape, high light energy utilization rate, organic combination of panicle number, panicle grain number, and 1000-grain weight. **(B)** Hi-C heat map of BD8 chromosome interactions.

Prior to the genome assembly, genome size and heterozygosity rate of BD8 was surveyed with clean Illumina pair-end short reads by k-mer analysis. Genome size was estimated to be approximately 387.76 Mb with a heterozygosity rate of 0.17%. Subsequently, PacBio HiFi reads (high accuracy) and ONT ultra-long reads (long-range continuity) were co-assembled using HiFiasm, generating 453 initial contigs with a contig N50 of 31.69 Mb. Illumina short reads were used for error correction. Valid Hi-C reads were aligned to the contig assembly to construct chromosome-level scaffolds using Lachesis. The interaction heatmap showed strong intra-chromosomal interactions and clear inter-chromosomal boundaries, confirming proper scaffolding (**Figure 1B**). Finally, chromosome orientation was adjusted against the Nip-T2T reference, and manual curation resolved misjoins. At completion, the confirmed genome size of BD8 was 384.27 Mb with 12 Chromosomes containing 12 centromeres, 23 telomeres (**Table 1**).

**Table 1:** Statistics of genome assembly and annotation for BD8 and Nip-T2T.

Genomic features	BD8	Nip-T2T
Assembly		
Total length of assembly (bp)	384,274,281	385,710,679
Number of contigs across Chromosomes	12	12
N50 of contigs across Chromosomes (bp)	31,688,881	31,111,469
Telomere number	23	24
Centromere number	12	12
GC%	43.57%	43.72%
Quality		
Genome BUSCO (%)	98.88	98.27
Number of major gaps	0	0
Number of unplaced contigs	0	0
Protein BUSCO (%)	98.76	97.36
QV	51.04	62.80
LAI	22.20	22.11
Annotation		
Number of protein-coding genes	58,685	57,359
Average gene length (bp)	2796	2941
Average CDS length (bp)	1175	1330
Number of TEs	413,234	320,909
Number of rRNA genes	1,294	1,747
Repeat sequences (%)	58.31	51.08

### The BD8 assembly exhibited high accuracy and completeness

Multiple criterions had been applied to evaluate the accuracy and completeness of BD8 assembly. Above all, the completeness of this BD8 assembly was evaluated by BUSCO (Manni et al., 2021)(Benchmarking Universal Single-Copy Orthologs), and 98.95% (1597) of core genes in the embryophyta_odb10database (1614), including 1557 (96.47%) single-copy complete BUSCOs and 40 (2.48%) complete and duplicated BUSCOs (**Supplementary Tables S2**), were completely verified, which is comparable to two gold standard rice reference assemblies, the *Japonica* variety *Nipponbare* (*Nip*) (98.9%) (Shang et al., 2023) and the *Indica* variety *Shuhui498* (*R498*) (98.6%) (Du et al., 2017). Subsequently, the primary data, including HiFi reads, ONT reads and Illumina short reads, was remapped to BD8 assembly with high mapping rates and 99.39% of Illumina reads, 99.42% of HiFi reads and 99.20% ONT reads were anchored to the final assembly, respectively (**Supplementary Table S3**). These results implied that the genome assembly of BD8 was performed well and in high completeness. Meanwhile, Qv (quality value), which was acquired by Merqury (Rhie et al., 2020), was also used to appraising genome accuracy. The Qv value (51.04) of this BD8 assembly signified high accuracy. The high LAI score (22.20), which evaluates the proportion of intact LTR-RT (Long Terminal Repeat Retrotransposons) in this BD8 assembly, demonstrated that the assembly reached the “gold standard” level (LAI >20) (Ou et al., 2018). In total, this genome assembly achieves high continuity, integrity, and accuracy, indicative of a high-quality rice reference genome.

### Annotation of BD8 assembly provided comprehensive genome information

Telomeres are an important part of chromosome, and could protect chromosome from damage and degradation during replication. Using seven-base telomeric repeats (“AAACCCT”) or (“AGGGTTT”) as a sequence query, most telomeres except on chromosome 9 short arm were detected in this BD8 assembly, with hundreds to thousands of copies of query sequence (**Supplementary Table S4**). The telomeres span from 5,446 bp to 11,117 bp, with an average length of 7,394 bp and a cumulative length of 81,331 bp. Centromeres, which are essential for ensuring accurate chromosomal segregation during cell division, are usually composed of abundance of retrotransposon and tandem repeats (Brar and Amon, 2008). Similarly, all 12 centromeres were identified in this long read-based assembly and the average length of the 12 centromeres is 518.99 kb, with the longest centromere observed on Chr01 (949.59 kb) and the shortest on Chr05 (118.29 kb) (**Supplementary Table S4**). Notably, no significant correlation was observed between centromere length and chromosome size.

As repetitive sequences usually constitute large proportions of rice genome and often play important roles in genome size variation and functional adaption, TEs and other repetitive sequences of the BD8 assembly were annotated before gene annotation. Repetitive elements were identified and annotated both using *de novo* and homology-based repeat approaches. As a result, 413,234 TEs (46.87%, 180,106,561 bp in total length) and 215,750 tandem repeat sequence (4.73%,18,193,690 bp in total length) were identified, which totally accounts for 51.60% of this BD8 assembly (**Supplementary Table S5**). It is higher than that in the genome of *O. sativa* (40.43% in Nip and 42.05% in R498). Among these repetitive sequences, DNA transposons were the most abundant, occupying 29.48% of the genome, followed by long terminal repeats (LTRs; 16.63%) (**Supplementary Table S5**). Indeed, this higher proportion of DNA transposons in BD8 is the most important contributors to the genome size variation.

**Figure 2:**
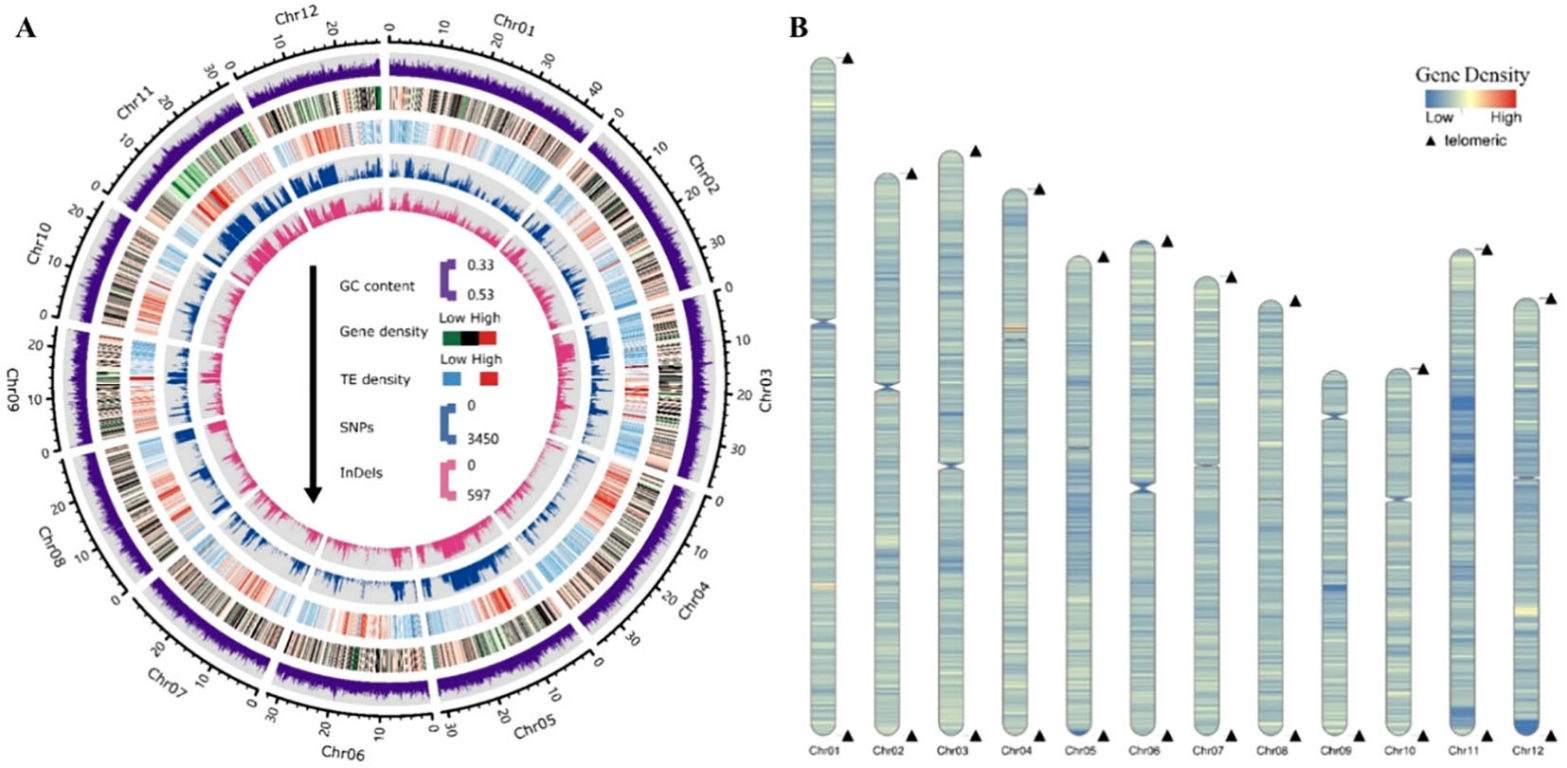
Overview of BD8’s genome feature. **(A)** Circos plot of gene features at 100-kb intervals across the 12 chromosomes of the genome of BD8. The GC content, gene density, TE density, and SNPs and InDels between the BD8 and Nip-T2T genomes are shown (from the outerring to the innerring). The outer black track represents the chromosomes of the genome assembly (with units in Mb). **(B)** Ideogram showing the distribution of predicted genes.

After excluding the repetitive sequences on the BD8 assembly, gene structure annotation was performed using a combination of *de novo*, homologous, and transcript prediction methods. As a result, a total of 58,685 protein-coding genes (162,177,958 bp in total length) were annotated and 87.99% (51,634) of genes are supported by at least two prediction methods, indicating high prediction quality (**Supplementary Table S6**; **Supplementary Figure S1**). The average gene and coding sequence lengths were 2763 bp and 1456 bp, respectively, with an average of 4.06 exons per gene (**Table 1**; **Supplementary Table S7**). Among these protein-coding genes, 95.93% (56297) could be annotated by at least one of the eight functional databases, including NR, eggNOG, GO, KEGG, TrEMBL, KOG, SWISS-PROT and Pfam (**Supplementary Table S8**). The genes were unevenly distributed along the chromosomes with an average density of 15.71 genes per 100Kb, and were sparse in the region of centromeres (**Figure 2A**). In addition, 3199 noncoding RNAs and 272 pseudogenes were identified. Among those potential noncoding RNAs, it includes 1294 ribosomal RNAs (rRNAs), 797 transfer RNAs (tRNAs), 812 microRNAs (miRNAs), 136 small nuclear RNAs (snRNAs) and 160 small nucleolar RNAs (snoRNA) (**Supplementary Table S9**).

### Comparative genomic analysis reveals BD8’s relationship and unique gene families

**Figure 3:**
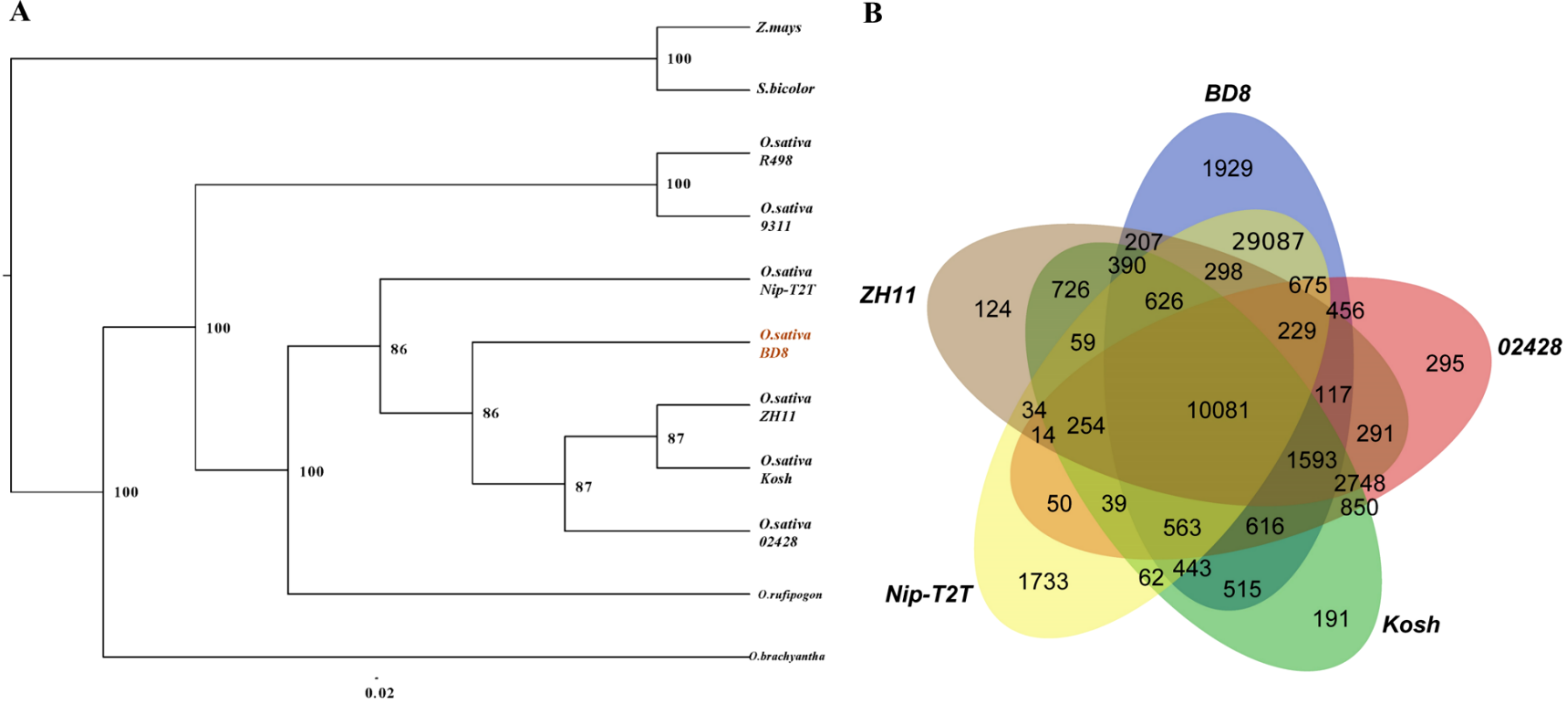
Relationship between BD8 and interspecific and intraspecific assemblies. **(A)** Phylogenic tree of representative interspecific and intraspecific assemblies together with BD8. The number indicated branch support values. The right is species/varieties name (**Supplementary Table S10**), and the bottom is the scale of evolutionary distance. **(B)** Veen diagram for gene families in *Japonica* assemblies.

Representative interspecific and intraspecific assemblies (**Supplementary Table S10**) together with BD8 were selected for identifying homologous genes, conducting gene family clustering, and constructing phylogenetic tree. A total of 367,498 annotation genes from those assemblies were extracted and incorporated into 59,374 gene families. Comparisons of genome synteny between BD8 and other assemblies have provided a framework to reveal evolutionary processes that lead to diversity of genome structure and function in many lineages. The phylogenetic tree was constructed basing 2066 single copy lineal homologous gene, which used the *Z. mays* and *S. bicolor* as out-group. It indicated that BD8 was clustered to the clade of *O. sativa* subsp. *Japonica* with five *Japonica* assemblies (*BD8, Nip-T2T, 02428, ZH11 and Kosh*), and had the closest genetic relationship with *ZH11* and *02428* (**Figure 3A**). Comparison of the gene families among those assemblies in this clade, 18,794 (51.5%) core gene families are commonly conserved in the five genomes, whilst 1929 unique gene families are present only in BD8 (**Figure 3B**).

### Genome-wide comparison between BD8 and Nip-T2T uncover BD8’s abundant genetic variation

Genome-wide collinear comparison was performed between BD8 assembly and reference genome Nip-T2T. Overall, the BD8 assembly (384.27 Mb) was 1.44 Mb shorter than Nip-T2T (385.71 Mb) (**Table 1**). The relative lengths of most BD8 chromosomes are approximately consistent with Nip (difference<1 Mb), except chromosome 9 of which BD8 is 3.71 Mb shorter than Nip-T2T due to the absence of telomere at short arm (**Supplementary Table S12**). Further comparison genome composition, BD8 contains less transposable elements sequences (180.11 Mb in BD8, 188.80 Mb in Nip-T2T) and less encoding sequence length (162.18 Mb in BD8, 168.39 Mb in Nip-T2T), resulting in a shorter genome size (**Supplementary Table S10**). Comparing genes homology with each other, BD8 shared 46444 (79.14%) unique homologous genes with Nip-T2T.

Subsequently, BD8’s genomic variations were further explored through directly alignment with Nip-T2T reference genome. It revealed a total of 611,287 SNPs, 126,780 InDels, with an average density of 1.59 SNPs and 0.33 InDels per kb, respectively. Among them, 344,923 SNPs and 72,914 Indels located in the genic region (9,795 genes) and 87,326 SNPs and 6,026 Indels located in coding regions (6,971 genes) (**Supplementary Table S13**). The distribution of SNPs/Indels was uneven across different chromosomes and within a chromosome. For example, on chromosome 11, SNPs and InDels were dense from 13.9 to 17.2 Mb, but sparse from the regions of 1.1-6.0 Mb and 17.3-20.2 Mb (**Figure 2B**).

**Figure 4:**
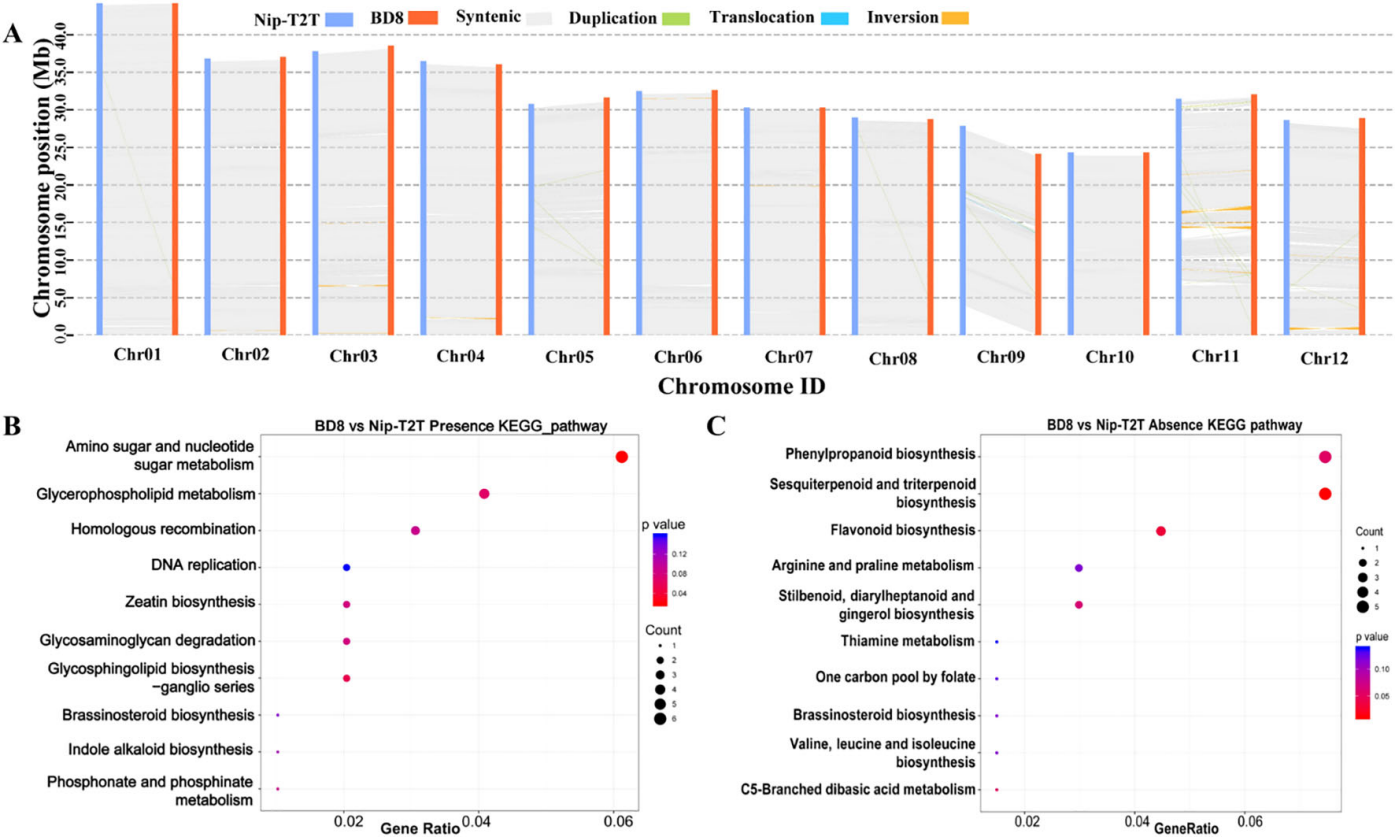
Characterization of genomic variations between BD8 and Nip-T2T. **(A)** Syntenic blocks shared between BD8 and Nip-T2T. Gray lines connect matched gene pairs. Inversion blocks are highlighted in orange. The translocation and duplication blocks are highlighted in light green and blue, respectively. **(B)** Enriched KEGG pathways from genes present in BD8 genome assembly. **(C)** Enriched KEGG pathways from genes absent in BD8 genome assembly.

It is also noteworthy that SNPs and InDels are always not enough to capture all the meaningful genomic variations that underlie crop improvement, and structure variants (SVs) also play an important role in genomic variations (Wellenreuthe et al., 2019). A total of 9016 Structure Variation (SVs) were detected between *BD8* and *Nip-T2T*, including 907 Present Variation (PV), 876 Absent Variation (AV), 33 Inversion Variation (INV), 485 Chromosomal Translocation Variation (CTV), 6715 Copy Number Variation (CNV) (**Supplementary Table S14**). The most remarkable difference between the two genomes was a 736,138bp inter-chromosomal translocation near short arm telomere on chromosome 12 of BD8, which located on chromosome 11 of Nip-T2T reference genome (**Figure 4A**). Those annotated genes, which were fully contained in the Present and Absent Variation (PAVs), were addressed. Five hundred eleven genes were present (235) or absent (276) between the two genomes owning to those PAVs. The genes present in BD8 enriched in only one significant KEGG pathways including amino sugar and nucleotide sugar metabolism (ko00520) (**Figure 4B**; **Supplementary Table S15**). Correspondingly, the genes absent in BD8 enriched in three significant KEGG pathways, including phenylpropanoid biosynthesis(ko00940), sesquiterpenoid and triterpenoid biosynthesis (ko00909), and flavonoid biosynthesis (ko00941) (**Figure 4C**; **Supplementary Table S15**).

### Time-course transcriptomic analysis provides insight into BD8 responses to salt stress

**Figure 5:**
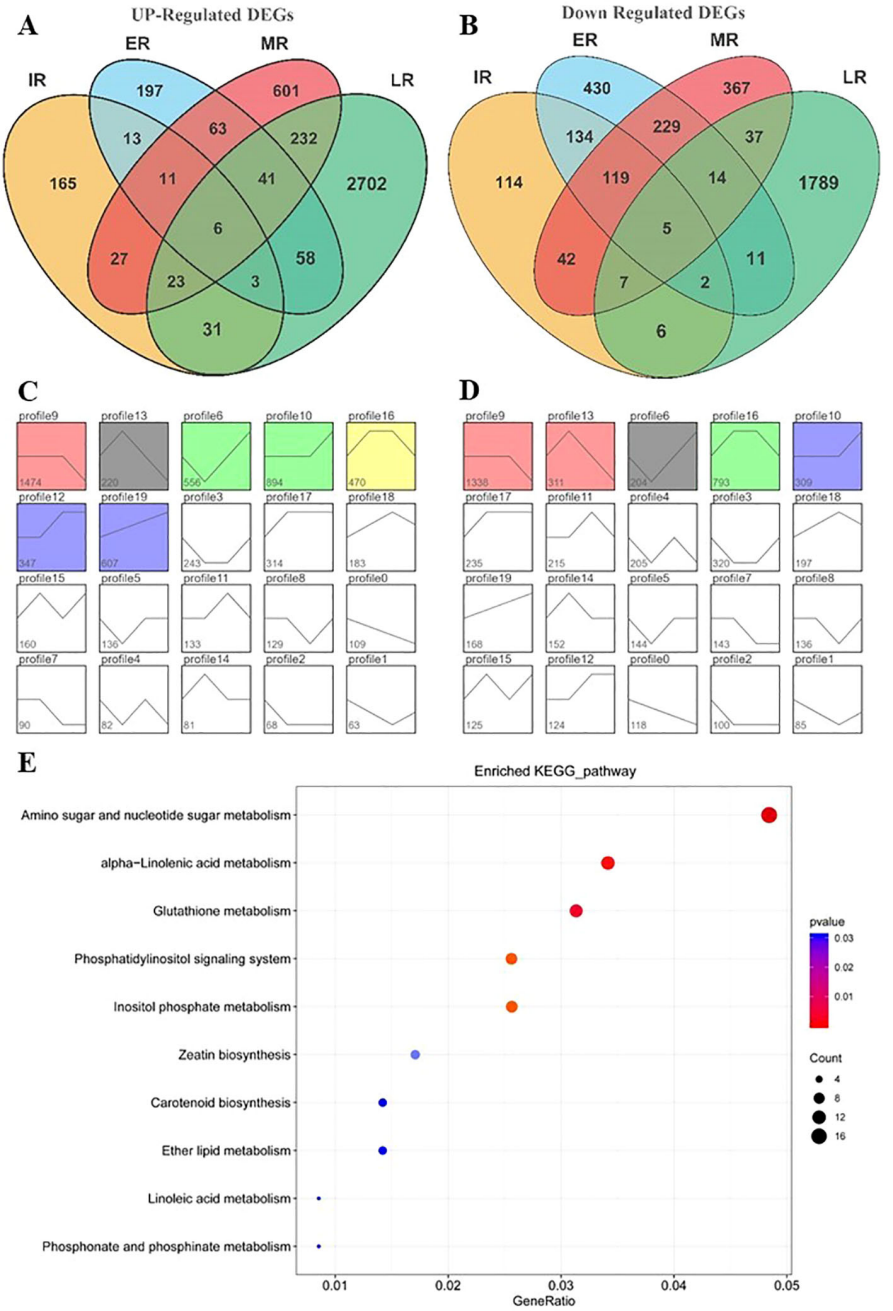
Time-course transcriptomic feature of BD8 responses to salt stress. **(A)** Up-regulated BD8’s DEGs under salt stress stages. **(B)** Down-regulated BD8’s DEGs under salt stress stages. **(C)** Trend analysis of the BD8’s DEGs under salt stress. **(D)** Trend analysis of the BD8’s DEGs under normal condition. **(E)** Kyoto Encyclopedia of Genes and Genomes (KEGG) diagrams of BD8’s DEGs in salt special enriched profiles.

To explore BD8’s salt-tolerant mechanism and identify its specific pathways in response to salt stress, time-series transcriptomic changes of BD8 were profiled by RNA-seq at initial (IR, initial stage RNA-seq, 1h),early (ER, early stage RNA-seq, 12 h), middle (MR, middle stage RNA-seq, 48 h), and late (LR, late stage RNA-seq, 96 h) salt stress stage, and four corresponding control group without stress, with each group consisting of three biological replicates. Compared with normal condition, 708, 1336, 1824 and 4967 differentially expressed genes (DEGs) were identified in BD8 at the initial, early, middle, and late salt stress stage, respectively (**Supplementary Figure S3**). The incremental number of DEGs between salt and normal condition suggested that the gene expression patterns were constantly changing during the four stages of salt stress. In the pursuit of identifying BD8’s pivotal salt-tolerant genes, trends of all those DEGs across four stages were analyzed. When delving deeper into the regulated direction of genes across stress stages, 4173 up-regulated DEGs, and 3506 down-regulated DEGs were obtained (**Figures 5A, B**). As merging them together, the DEGs were clustered into 20 profiles by time-course trend analysis. Comparing the significant enriched profiles between stress and control condition, the Profile 12 and 19 were specially enriched under salt stress condition and were highlighted. The DEGs in those profiles showed constantly up-regulated trend containing 347 (Profile12) and 607 (profile19) respectively, which were considered as BD8’s salt-special response genes. It indicated that those genes enriched into five KEGG pathways, including alpha-Linolenic acid metabolism (ko00592), Amino sugar and nucleotide sugar metabolism (ko00520), Glutathione metabolism (ko00480), Phosphonate and phosphinate metabolism (ko00440), Ether lipid metabolism (ko00565). Notably, the pathway of Amino sugar and nucleotide sugar metabolism (ko00520) was commonly enriched from BD8’s special present genes and salt-specific gene clusters. To confirm the accuracy and reproducibility of DEGs identified using RNA-seq, the expression levels of six randomly selected genes in the pathway ko00520 were verified with qRT-PCR analysis, and the relative expression trends showed general consistency between the two methods (**Supplementary Figure S4**).

## Discussion

High-quality genome assemblies are foundational for dissecting crop genetic diversity and accelerating molecular breeding (Varshney et al., 2021), yet the “dark genome” regions (e.g., centromeres, telomeres) remain challenging to resolve due to their repetitive nature (Rhie et al., 2021). Here, we present the first near complete genome of a North China Japonica rice variety, BD8, leveraging HiFi and ONT ultra-long reads to achieve a gap-free assembly with a contig N50 of 31.69 Mb—comparable to the gold-standard Nip-T2T genome (Shang et al., 2023). This assembly not only fills the gap in genomic resources for regional rice varieties but also provides a critical reference for deciphering structural variations (SVs) and gene functions underlying adaptive traits like salt tolerance.

The integration of HiFi (high accuracy) and ONT ultra-long (long-range continuity) reads enabled precise assembly of BD8’s centromeres and telomeres, with only one missing telomere (Chr9 5’ end)—likely addressable via deeper ONT sequencing. This completeness is pivotal, as centromeric and subtelomeric regions often harbor stress-responsive genes and SVs (Garg et al., 2024). For instance, the 12 fully resolved centromeres in BD8 (each containing CentO satellite repeats) provide a framework to study karyotype evolution and centromere function in Japonica rice, which has diverged from indica varieties in centromere structure (Shang et al., 2022).

While over 3,000 rice genomes are deposited in the China National Center for Bioinformation (https://ngdc.cncb.ac.cn/gwh), most are draft assemblies, with only seven achieving telomere-to-telomere resolution and annotation. BD8’s genome expands this limited set of telomere-to-telomere rice assemblies (e.g., Nip, R498), underscoring the necessity of diverse regional varieties to capture pan-genomic complexity. Given that core genes account for <20% of the rice pan-genome (Qin et al., 2021), BD8’s genome—enriched with North China-specific genetic variations—will facilitate the mining of novel alleles for stress tolerance and yield improvement.

Annotation of BD8’s genome identified 58,685 genes, 88.16% supported by multiple prediction methods, ensuring reliability for downstream functional studies. Comparative analysis with Nip-T2T revealed abundant SVs, including 235 present and 276 absent genes (PAVs). These PAVs enriched in pathways like amino sugar/nucleotide sugar metabolism (ko00520) and phenylpropanoid biosynthesis (ko00940), which are linked to cell wall modification and secondary metabolism—key for biotic/abiotic stress responses (Zhou et al., 2023). Notably, SVs often have larger phenotypic impacts than SNPs/InDels (Zhang et al., 2022), making BD8’s PAVs prime candidates for explaining its unique salt tolerance and environmental adaptability in North China.

Time-course transcriptomics under salt stress identified two BD8-specific gene clusters (Profile12/19) with 511 upregulated genes, enriching pathways like glutathione metabolism (osmoprotection) and alpha-linolenic acid metabolism (jasmonate signaling). Strikingly, amino sugar/nucleotide sugar metabolism (ko00520) was commonly enriched in both BD8-specific PAVs and salt-responsive clusters, highlighting it as a potential hub for salt tolerance. This pathway regulates cell wall integrity (via UDP-sugars) and osmotic balance (via compatible solutes like trehalose), mechanisms previously linked to salt tolerance in rice (Kishor et al., 2005). For example, upregulation of UDP-glucose 4-epimerase (UGPase) in BD8 under salt stress may enhance cell wall rigidity by promoting cellulose synthesis, reducing Na+ influx. Future studies should validate key genes in this pathway (e.g., UGPase, GMPase) via CRISPR editing to confirm their role in BD8’s salt tolerance.

BD8’s near telomere-to-telomere genome provides a high-resolution map for genomics-driven breeding in North China. The identification of salt-responsive genes and SVs offers targets for marker-assisted selection (MAS), while the annotated centromeres/telomeres enable studies on chromosomal stability during meiosis—critical for hybrid rice development. Moreover, as a regional elite variety, BD8’s genome bridges the gap between pan-genomic resources and local breeding needs, facilitating the introgression of salt tolerance and yield-related alleles into adapted cultivars.

Despite its completeness, BD8’s genome warrants further investigation into the functional roles of PAVs and non-coding RNAs in salt tolerance. Integrating metabolomics with transcriptomics could reveal how amino sugar metabolism interacts with other pathways (e.g., flavonoid biosynthesis) to enhance stress resilience. Additionally, comparative analysis with other telomere-to-telomere genomes (e.g., indica varieties) will clarify the evolutionary dynamics of centromeres and stress-responsive loci in Oryza.

## Conclusion

In conclusion, BD8’s telomere-to-telomere genome assembly not only advances our understanding of rice genomic diversity but also provides a blueprint for dissecting adaptive traits and accelerating molecular breeding in regionally important crops. The convergence of structural variations and transcriptional responses in amino sugar metabolism underscores the power of integrating genomics and transcriptomics to unravel complex stress tolerance mechanisms.

## Data Availability

The raw sequencing data have been deposited in NGDC with the BioProjectID PRJCA038088 and BioSample accession: SAMC4946132. The BD8 assembly number is GWHFWBB00000000.1.

## Supplementary material

Supplementary Figure 1 Field growth and root system performance of BD8 under salt stress (6 ms/cm).

Supplementary Figure 2 Number of gene annotation by ab initio, homolog, and transcriptome prediction methods, respectively.

Supplementary Figure 3 Differentially expressed genes (DEGs) were identified in BD8 at the initial, early, middle, and late salt stress stage after salt stress, respectively.

Supplementary Figure 4 Gene expression changes under salt stress and normal conditions at late salt stress stage based on RT-qPCR and RNA-seq.
