# Supplementary figures and images for: A gapless genome assembly of a Japonica variety ‘BD8’ provides insights into rice salt tolerance
Source: Front Plant Sci. 2025 Dec 12;16:1713117. doi: 10.3389/fpls.2025.1713117 (PMC12741066; doi:10.3389/fpls.2025.1713117)

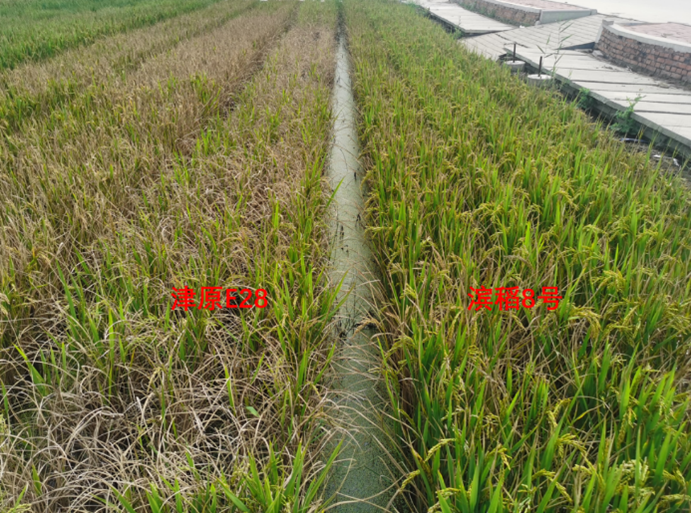

Supplement: Supplementary Figure 1 — Field growth and root system performance of BD8 under salt stress (6 ms/cm). [file Image1.tif]

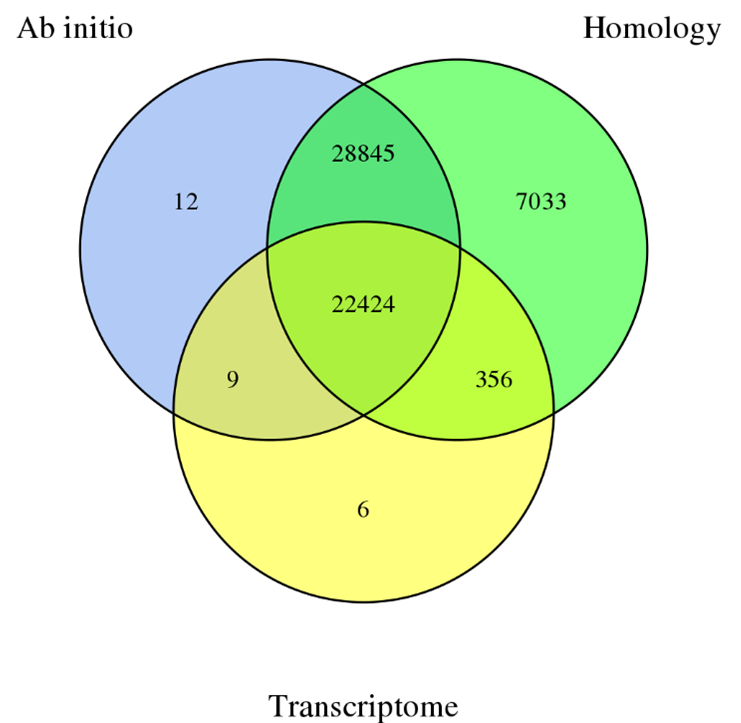

Supplement: Supplementary Figure 2 — Number of gene annotation by ab initio, homolog, and transcriptome prediction methods, respectively. [file Image2.tif]

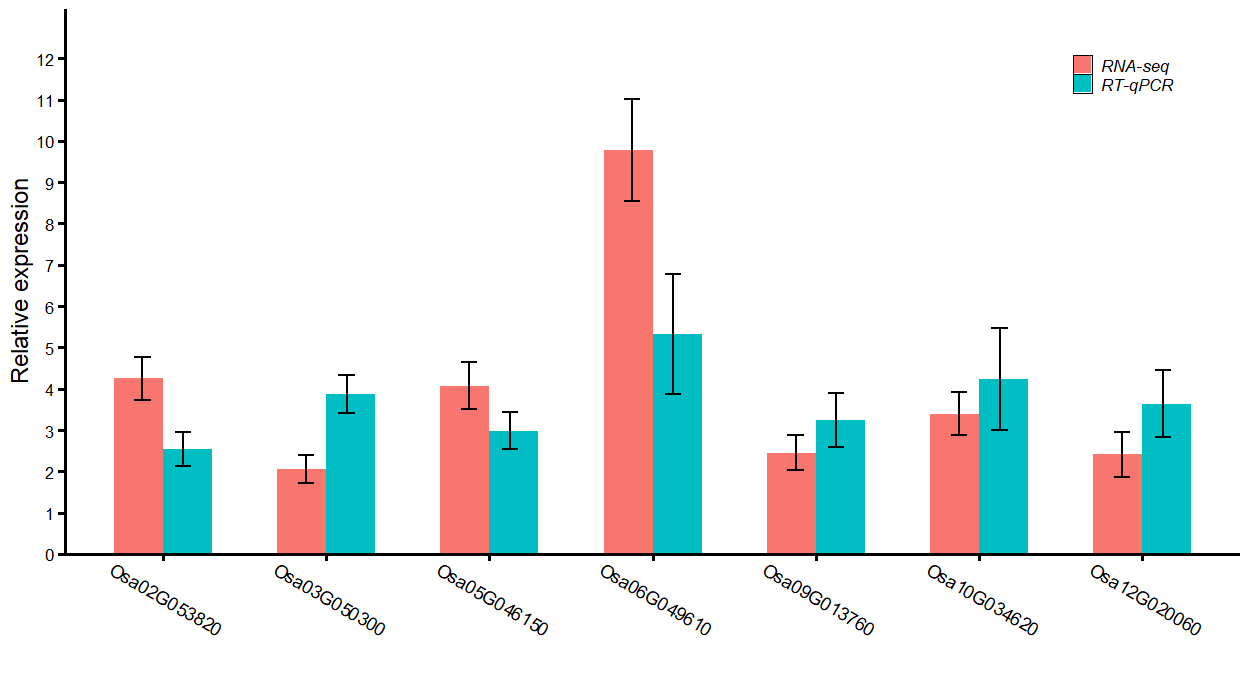

Supplement: Supplementary Figure 3 — Differentially expressed genes (DEGs) were identified in BD8 at the initial, early, middle, and late salt stress stage after salt stress, respectively. [file Image3.tiff]

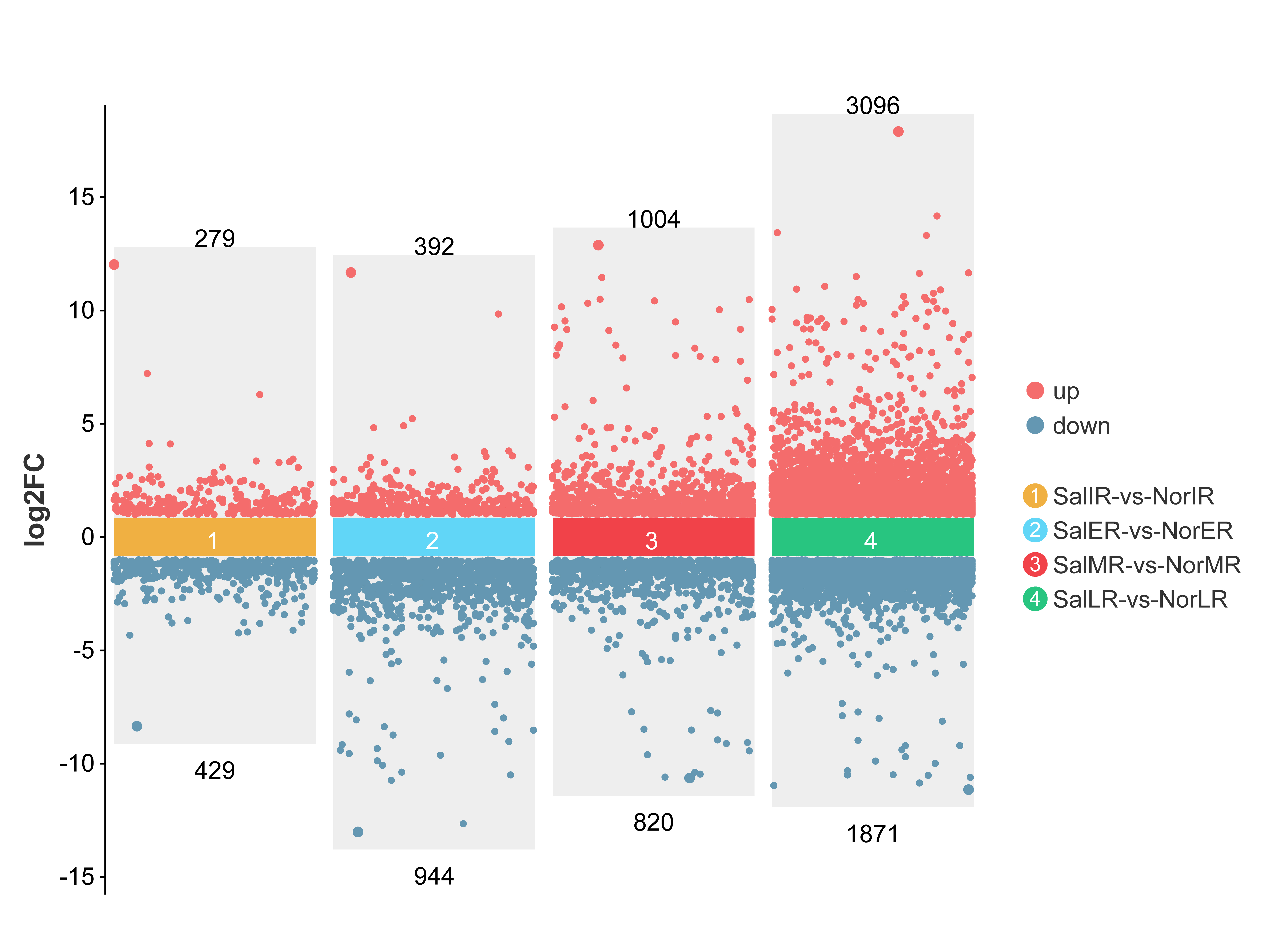

Supplement: Supplementary Figure 4 — Gene expression changes under salt stress and normal conditions at late salt stress stage based on RT-qPCR and RNA-seq. [file Image4.jpeg]
